# Prognostic value of LncRNA PSMA3-AS1 in prostate cancer and its potential regulatory mechanism

**DOI:** 10.1186/s41065-025-00485-6

**Published:** 2025-07-12

**Authors:** Muyang Cao, Jin Li, Jianbin Zhang, Wenlong Lu

**Affiliations:** 1 Department of Urology, Harbin Medical University Cancer Hospital, Harbin, 150081 China; 2 Department of Urology, the NO.983 Hospital of The People’s Liberation Army Joint Logistic Support Force, Tianjin, 300142 China; 3 Department of Urology, Shanxi Hospital Affiliated to Cancer Hospital, Shanxi Province Cancer Hospital, Chinese Academy of Medical Sciences, Cancer Hospital Affiliated to Shanxi Medical University, Taiyuan, 030001 China; 4 Department of Urology, Shanghai Fengxian District Central Hospital, No.6600 Nanfeng Road, 201499 Shanghai, China; 5 Department of Urology, Sixth People’s Hospital South Campus Affiliated to Shanghai Jiaotong University, No.6600, Nanfeng Highway, Fengxian District, Shanghai, 201499 China

**Keywords:** Prostate adenocarcinoma, Prognostic, PSMA3-AS1, miR-29a-3p

## Abstract

**Objective:**

Prostate adenocarcinoma (PRAD) is asymptomatic in the early stages and most patients are diagnosed at an advanced stage, which leads to a poor prognosis. Therefore, an effective prognostic marker is required to improve PRAD prognosis.

**Methods:**

A total of 128 patients with PRAD were included in the study. PSMA3-AS1 and miR-29a-3p expression in tissues was detected using RT-qPCR. CCK-8 and Transwell assays were then used to evaluate the proliferative, migratory, and invasive capacities of prostate cancer cell lines. A DLR assay confirmed the binding relationship between PSMA3-AS1 and miR-29a-3p. The five-year prognosis of PRAD patients was analyzed using a Kaplan–Meier plotter curve.

**Results:**

PSMA3-AS1 was highly expressed in PRAD tissues, and patients with high expression had poor 5-year survival. In contrast, miR-29a-3p was poorly expressed in PRAD tissues. PSMA3-AS1 bound to miR-29a-3p in a targeted manner and the levels showed a negative correlation. Knocking down PSMA3-AS1 could increase the level of miR-29a-3p and slow the proliferation of PRAD cell lines, as well as inhibiting their migration and invasion ability.

**Conclusion:**

A high level of PSMA3-AS1 was strongly linked to a poor prognosis for patients and is expected to serve as a prognostic marker for PRAD. Furthermore, PSMA3-AS1 knockdown increased the level of miR-29a-3p and reduced the physiological activity of cancer cells. Therefore, regulating the expression of the PSMA3-AS1/miR-29a-3p axis could influence PRAD development.

**Supplementary Information:**

The online version contains supplementary material available at 10.1186/s41065-025-00485-6.

## Introduction

The development of cancer therapy has a long history. In modern times, the development of therapies targeting genes with oncogenes and associated signaling pathways, based on advances in molecular biology, is an important part of the evolution of cancer therapy [1]. Prostate adenocarcinoma (PRAD) is the most common malignant tumor of the male genitourinary system. In the early stage, there are no obvious symptoms [2–4]. As the disease progresses, symptoms such as urinary urgency, frequency and incontinence appear, which endanger the patient’s life [5]. The number of PRAD cases in 2022 will be the highest among male tumors (27%), and the number of deaths will be the second highest among male tumors (11%), second only to lung cancer [6]. Despite the availability of screening for PRAD, one of the most common malignancies in older men, the majority of patients present with a high risk or locally advanced disease at the time of initial diagnosis, missing the optimal treatment period. This results in a poor prognosis for most patients, with poor overall five-year survival rates. In addition, most of the current treatments regarding tumors are based on surgery, radiotherapy [7] and chemotherapy [8], which have certain side effects [9]. Therefore, an effective biomarker is needed to improve the prognosis of PRAD patients. This can also provide potential therapeutic targets for PRAD and help promote the development of targeted therapy for PRAD [10].

LncRNAs are a class of RNAs that do not encode proteins but have biological functions [11]. They are often expressed in a tissue-specific context, and in some disease types, lncRNAs that are differentially expressed can serve as biomarkers for disease diagnosis and prognosis [12, 13]. PCA3 and MALAT-1 are currently reported to be aberrantly expressed in PRAD and are reliable lncRNAs for the diagnosis of PRAD [14]. LncRNA PSMA3-AS1 is highly expressed in many cancers, such as oral squamous cell carcinoma [15], oesophageal cancer [16], lung cancer [17], colorectal cancer [18], and glioma [19]. It is highly expressed in bladder cancer [20], which is a urological tumor. More importantly, the expression level of PSMA3-AS1 in PRAD was significantly higher in the GEPIA version 2 database (http://gepia2.cancer-pku.cn/#index, which is an upgraded version of GEPIA) [21]. However, the PSMA3-AS1 level in PRAD, which is also a urological tumor, and its prognostic value have not been reported. Based on the fact that PSMA3-AS1 is highly expressed in many cancers, we hypothesize that it also shows high expression levels in PRAD.

miR-29a-3p was reported to act as a tumor suppressor in prostate adenocarcinoma in a study by Xiao et al. [22]. Kong et al. reported that miR-29a-3p was poorly expressed in PRAD tissue samples [23]. The ENCORI database revealed a targeting relationship. However, the regulatory mechanism through which PSMA3-AS1 targets miR-29a-3p in PRAD remains unknown. Therefore, the aim of this study was to investigate the expression level of PSMA3-AS1 and its prognostic value for PRAD, as well as the potential regulatory mechanism of PSMA3-AS1 in PRAD through the regulation of the level of miR-29a-3p.

## Materials and methods

### Patient inclusion

128 patients with PRAD who underwent surgery from February 2017 to October 2019 in Shanxi Province Cancer Hospital were included. Inclusion criteria: (1) patients met the relevant criteria for a clinical diagnosis of prostate cancer, as well as undergoing rectal fingerprinting, prostate biopsy and other tests for clarification; (2) all patients were diagnosed with PRAD for the first time; (3) patients’ clinical baseline data were complete. Exclusion criteria: (1) a history of prostate surgery; (2) other malignant tumors; (3) chronic prostatitis; and (4) a history of prostate cancer treatment. The patients’ cancerous and precancerous tissues were collected and stored in a refrigerator at -80℃ for subsequent experiments. Patients who underwent surgery were followed up for five years via telephone or outpatient visits.

All subjects and their families signed an informed consent form, and the study was approved by the Ethics Committee of Shanxi Province Cancer Hospital.

### Cell culture and transfected

The PRAD cell lines LNCaP, DU145, VCaP and PC-3, as well as the human normal prostate epithelial cell line RWPE-1, were purchased from the Cell Collection of the Chinese Academy of Sciences (Shanghai, China). The VCaP, DU145 and RWPE-1 cells were grown in DMEM medium, while the LNCaP and PC-3 cells were grown in RPMI 1640 medium. Both media contained 10% FBS and 1% dual antibiotics (streptomycin and penicillin). All cells were cultured at 37 °C in an atmosphere containing 5% CO₂.

The miR-29a-3p inhibitor, empty vector oligonucleotides and the shRNA negative control (sh-NC) and sh-PSMA3-AS1 were purchased from RiboBio (Guangzhou, China). These were then transfected into the cells using the Lipofectamine 3000 kit (Invitrogen, USA).

### RT-qPCR

The frozen tissues were fully ground into a powder and completely lysed by adding Trizol reagent to release the RNA. The total RNA extracted from the tissues was then reverse transcribed into cDNA using a reverse transcription kit. The primers were then designed for qPCR experiments using a PCR instrument. The procedure was set as follows: pre-denaturation at 95℃ for 300s; denaturation at 95℃, annealing at 55℃, extension at 72℃ for the 20s in each session, and repeated for 40 cycles. GAPDH and U6 were used as internal references for PSMA3-AS1 and miR-29a-3p, respectively, and the levels of the two were calculated by the 2^−ΔΔCt^ method.

### Cell proliferation assay

The cells were cultured in 96-well plates and their proliferative ability was assessed using CCK-8 every 24 h. Three replicate wells were inoculated with each type of transfected cell and cultured for 24, 48 and 72 h. At the end of the culture period, the medium in the plates was removed and 10 µL of CCK-8 solution was added to each well. The plates were then incubated with CCK-8 at 37 ℃ for 2 h. After incubation, the absorbance value at 450 nm (OD 450) was determined using an enzyme marker.

### Transwell assay

The migratory and invasive properties of PRAD cell lines were assessed using the Transwell method. For the migration experiments, the Transwell system was used, with a serum-free medium in the upper chamber and a serum-containing medium in the lower chamber. The transfected cells (density of 1 × 10⁴) were inoculated in the upper chamber. After 36 h of incubation, the migrated cells were fixed with methanol and stained with crystal violet.

For invasion experiments, the upper chamber of the Transwell system contained serum-free medium and was pre-coated with Matrigel, while the lower chamber contained serum-containing medium. The subsequent steps were the same as for the migration experiments. Under a light microscope, the stained cells were counted in five randomly selected fields.

### Dual-luciferase reporter assay (DLR)

The ENCORI database identified the miR-29a-3p binding site in PSMA3-AS1. Based on this finding, the researchers constructed a PSMA3-AS1 wild-type recombinant plasmid (PSMA3-AS1-WT) and a PSMA3-AS1 mutant recombinant plasmid (PSMA3-AS1-MUT). According to the instructions in the Lipofectamine^®^ 3000 kit (Thermo Fisher, USA), the luciferase-carrying WT and MUT plasmids were then co-transfected alongside miR-NC and either the miR-29a-3p mimics or inhibitors into LNCaP and PC-3 cells. The transfection temperature was 37 ℃ and the transfection time was 48 h.

### Statistical analysis

The number of patients included was analyzed according to the Gpower statistical software and calculated with a 5% margin of error and 95% confidence interval with a statistical power of 80% and alpha = 0.05. 128 patients with PRAD who met the inclusion criteria were included after accounting for patient attrition. The total number of patients included in our cohort was greater than the statistical minimum sample size. Data analysis and visualization were performed using SPSS 23 and GraphPad Prism 9.0. Data are presented as the mean ± standard deviation. The correlation between PSMA3-AS1 and miR-29a-3p was analyzed using Pearson’s correlation. Statistically significant differences are indicated by *P* values of less than 0.05.

## Results

### Comparison of clinical data

There were no significant differences in terms of age, smoking history or drinking history between PRAD patients with different expressions of PSMA3-AS1. However, there were significant differences in differentiation, PSA, Gleason score, TNM and LNM (Table 1, *P* < 0.05).

**Table 1:** Comparison of clinical data of patients with different expression levels of LncRNA PSMA3-AS1

Indicators	Total	Low level of PSMA3-AS1 (*n* = 60)	High level of PSMA3-AS1 (*n* = 68)	*P*
Age, years				
< 70	55	29	26	0.249
≥ 70	73	31	42	
Drinking				
NO	50	23	27	0.874
YES	78	37	41	
Smoking				
NO	37	16	21	0.600
YES	91	44	47	
Tumor size				
≤ 5 cm	56	24	32	0.422
> 5 cm	72	36	36	
Differentiation				
well, moderate	80	45	35	**0.006**
poor	48	15	33	
PSA, ng/mL				
<7	69	44	25	**0.000**
7	59	16	43	
Gleason score				
<10	83	50	33	**0.000**
10–20	45	10	35	
TNM				
I-II	95	53	42	**0.001**
III	33	7	26	
LNM				
NO	75	45	30	**0.000**
YES	53	15	38	

### PSMA3-AS1 expression and its prognostic value

**Fig. 1:**
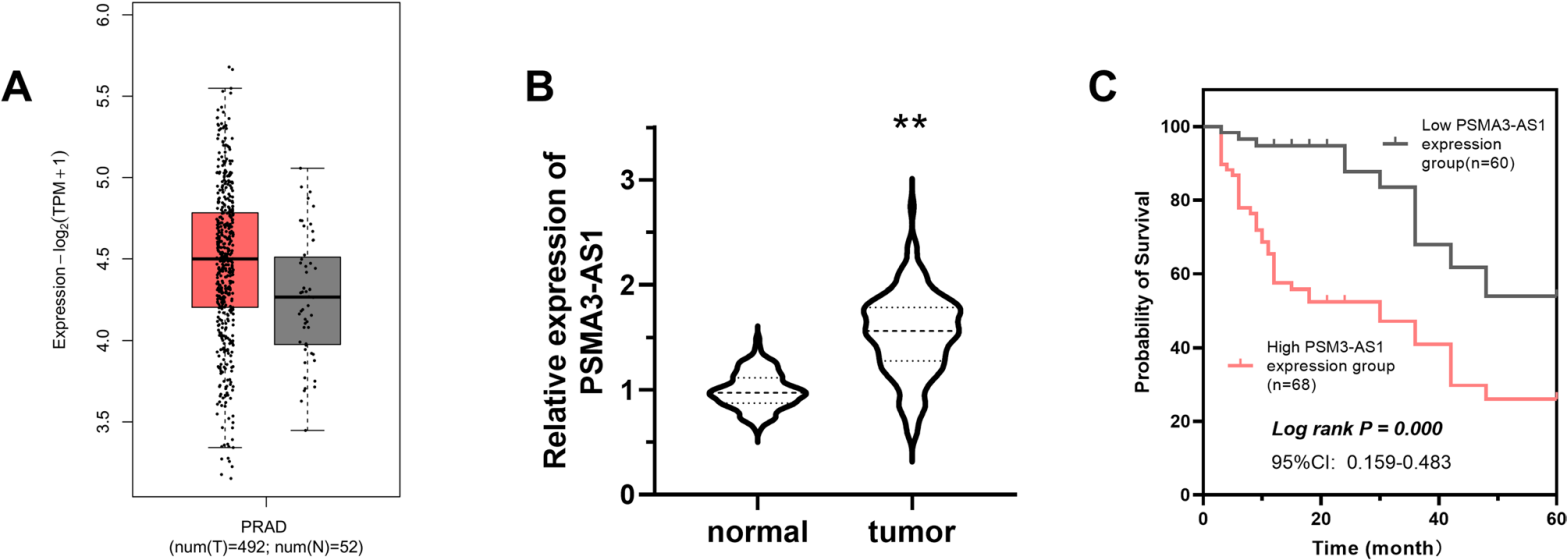
PSMA3-AS1 expression and its prognostic value. Database analysis revealed that PSMA3-AS1 was highly expressed in PRAD cancer tissues **(A)**. Our RT-qPCR also revealed that PSMA3-AS1 was notably upregulated in PRAD cancer tissues **(B)**. In addition, the Kaplan Meier curve results showed that patients in the PSMA3-AS1 high expression group had shorter 5-year prognostic survival **(C)**, (** *P* < 0.01 vs. normal)

The database results showed that PSMA3-AS1 was highly expressed in PRAD tissues compared to normal tissues (Fig. 1A, num[T] = 492, num[N] = 52). Our experiments had similar results, with PSMA3-AS1 significantly upregulated in PRAD (Fig. 1B, *P* < 0.01). In addition, the data of Kaplan-Meier curve indicated that the 5-year prognostic survival was shorter for patients in the PSMA3-AS1 high expression group (Fig. 1C, Log rank *P* = 0.000, 95%CI: 0.159–0.483). This suggests that PSMA3-AS1 is likely to be an independent influence on PRAD prognosis, as confirmed by the results of COX regression (*P* = 0.003, HR = 3.119, 95%CI: 1.467–6.633). In addition, the results of COX regression also indicated that PSA (*P* = 0.046, HR = 4.015, 95%CI: 1.027–15.707), Gleason score (*P* = 0.006, HR = 3.373, 95%CI: 1.413–8.053), TNM (*P* = 0.017, HR = 2.295, 95%CI: 1.158–4.548), and LNM (*P* = 0.004, HR = 3.637, 95%CI: 1.522–8.693) were also independent factors influencing the prognosis of PRAD (Table 2).

**Table 2:** Multivariate Cox analysis of PSMA3 -AS1 and clinical parameters with overall survival

Characteristics	Multivariate analysis		
	*P*	HR	95%CI
PSMA3 -AS1	0.003	3.119	1.467–6.633
Differentiation	0.102	2.639	0.826–8.432
PSA	0.046	4.015	1.027–15.707
Gleason score	0.006	3.373	1.413–8.053
TNM	0.017	2.295	1.158–4.548
LNM	0.004	3.637	1.522–8.693

### Effect of PSMA3-AS1 expression on prostate cancer cell lines

**Fig. 2:**
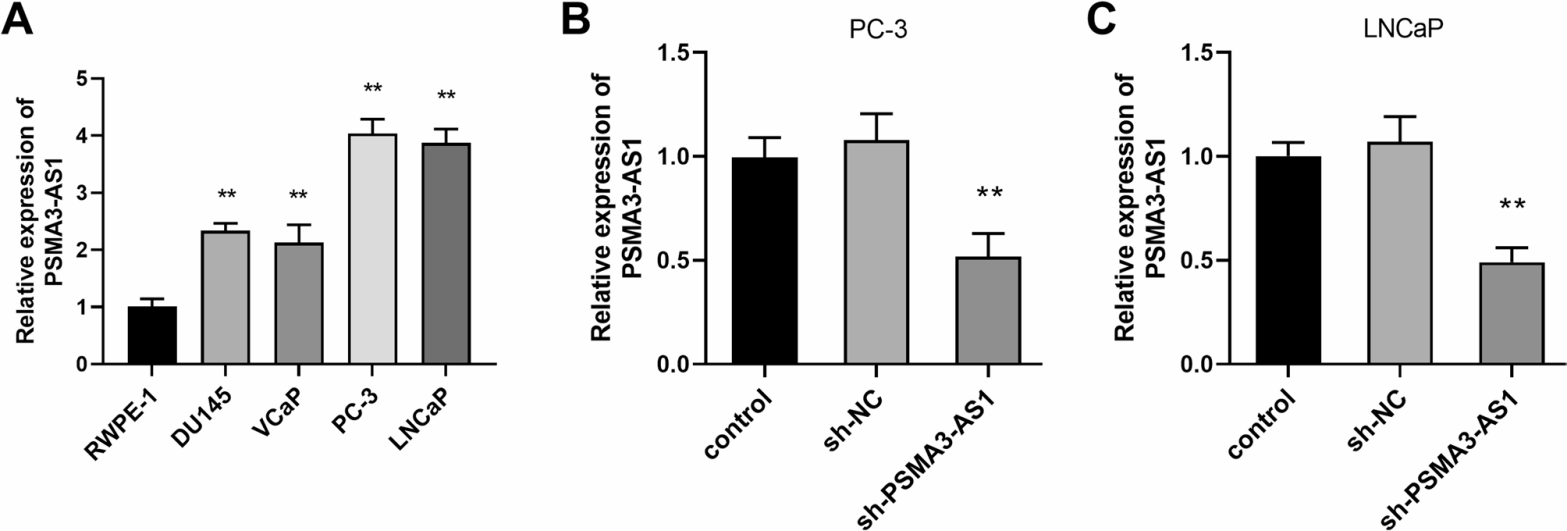
PSMA3-AS1 levels in PRAD cell lines. PSMA3-AS1 levels were significantly elevated in PRAD cell lines **(A)**. Knockdown of PSMA3-AS1 resulted in significantly lower PSMA3-AS1 expression levels in prostate cancer cell lines **(B)**, (** *P* < 0.01 vs. RWPE-1 or control)

**Fig. 3:**
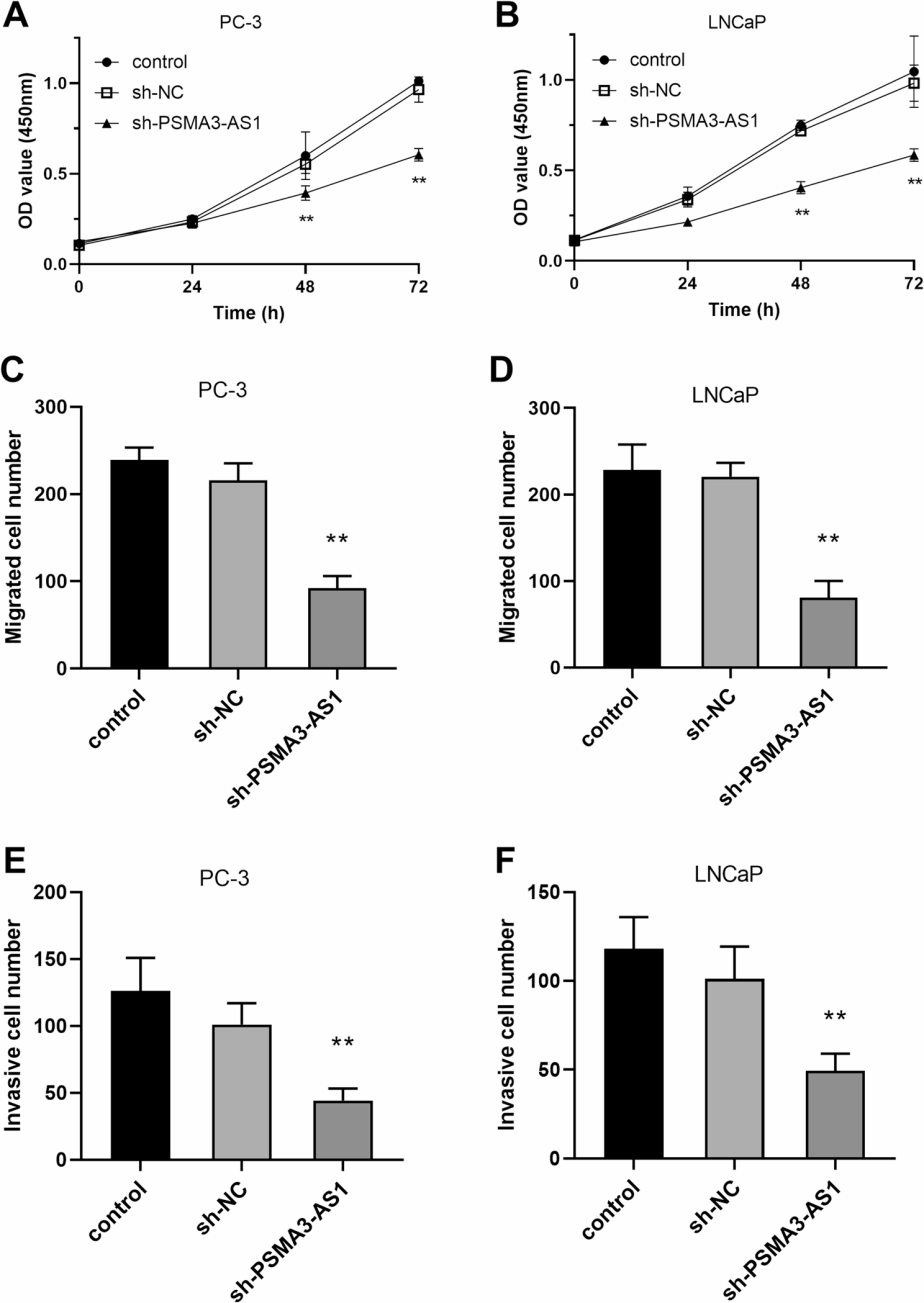
Effect of PSMA3-AS1 expression on PRAD cell lines. Knockdown of PSMA3-AS1 resulted in a significant reduction in the proliferative capacity of PRAD cell lines **(A and B)**. Knockdown of PSMA3-AS1 resulted in a significant reduction in the migration and invasion ability of PRAD cell lines **(C-F)**, (** *P* < 0.01 vs. control)

As illustrated in Fig. 2A, all PRAD cells showed a significantly higher level of PSMA3-AS1 expression compared to RWPE-1 cells (*P* < 0.01). As PSMA3-AS1 levels were higher in LNCaP and PC-3 cells, these two were selected for subsequent experiments. Knockdown of PSMA3-AS1 resulted in markedly lower levels of the gene in PRAD cells (Fig. 2B-C, *P* < 0.01). In addition, silencing PSMA3-AS1 level reduced the proliferative ability of LNCaP and PC-3 cells (Fig. 3A-B, *P* < 0.01). The migration and invasion of LNCaP and PC-3 cells showed the same trend, and silencing of PSMA3-AS1 led to a marked reduction in the migratory and invasive ability of PRAD cell lines (Fig. 3C-F, *P* < 0.01).

### Targeted binding of PSMA3-AS1 to miR-29a-3p

**Fig. 4:**
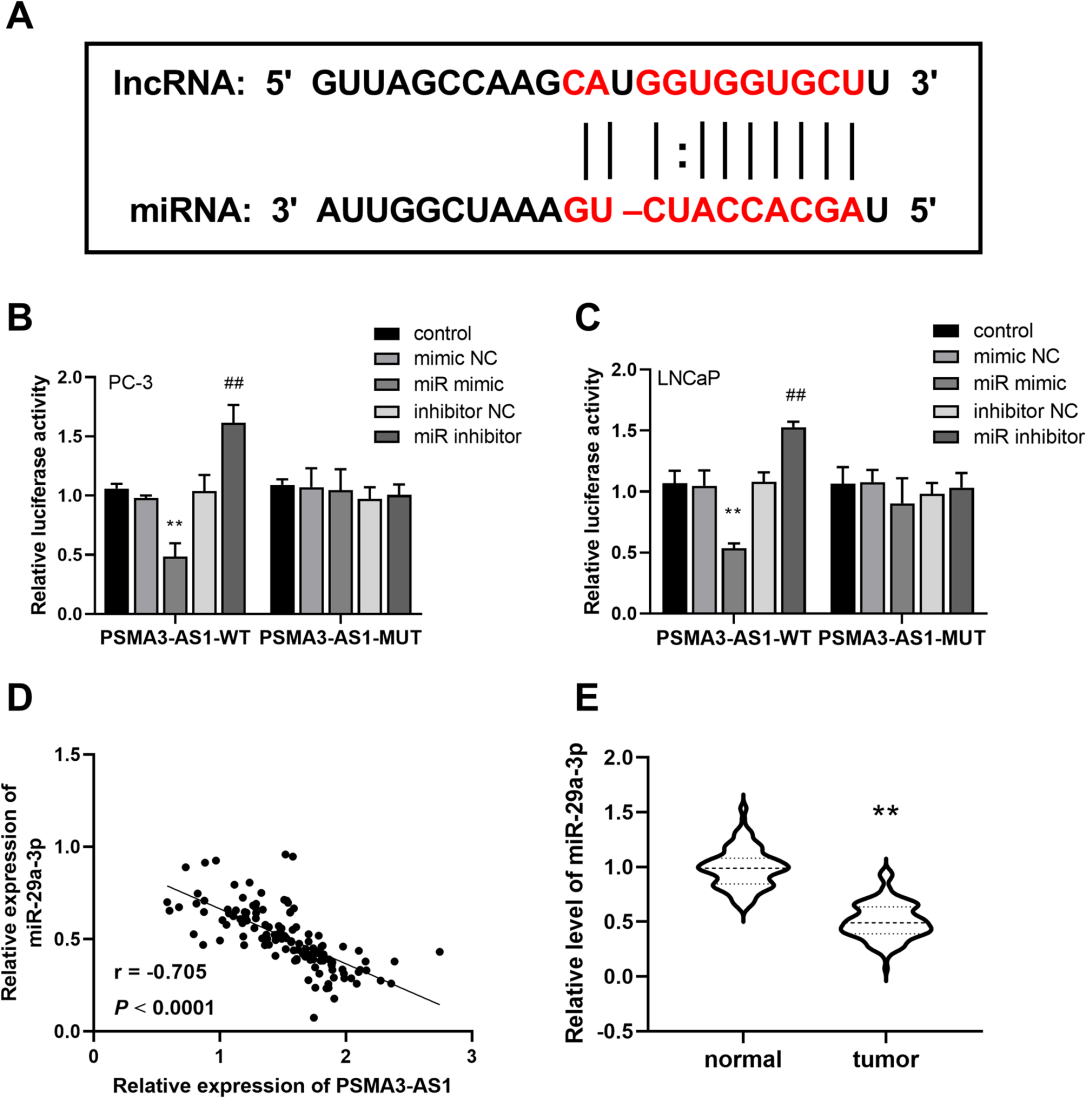
Targeted binding of PSMA3-AS1 to miR-29a-3p. Possible binding sites of PSMA3-AS1 and miR-29a-3p **(A)**. DLR experiments verified the binding relationship between PSMA3-AS1 and miR-29a-3p **(B-C)**. PSMA3-AS1 was negatively correlated with miR-29a-3p expression level **(D)**. In PRAD cancer tissues, miR-29a-3p was significantly reduced **(E)**, (** *P* < 0.01 vs. mimic NC or normal; ## *P* < 0.01 vs. inhibitor NC)

Figure 4A demonstrates the possible binding site of PSMA3-AS1 to miR-29a-3p. We verified the binding relationship using DLR experiments. The results showed that PSMA3-AS1-WT luciferase activity was significantly reduced when it was transfected with miR-29a-3p mimics, and significantly increased when miR-29a-3p was inhibited. These results suggest that PSMA3-AS1 binds to miR-29a-3p (Fig. 4B-C, *P* < 0.01). And PSMA3-AS1 exhibited a negative correlation with the miR-29a-3p expression level (Fig. 4D, *r*=-0.705, *P* < 0.01). And miR-29a-3p was remarkably reduced in PRAD cancer tissues (Fig. 4E, *P* < 0.01).

### PSMA3-AS1 and miR-29a-3p together affect cell physiological functions

**Fig. 5:**
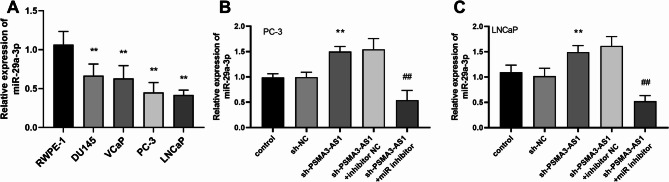
MiR-29a-3p expression level in PRAD cell lines. The miR-29a-3p expression level was significantly decreased in all PRAD cell lines **(A)**. In PRAD cell lines, knockdown of PSMA3-AS1 caused a significant increase in miR-29a-3p level; however, this effect was notably suppressed by the addition of miR-29a-3p inhibitor **(B-C)**, (** *P* < 0.01 vs. sh-NC; ## *P* < 0.01 vs. sh- PSMA3-AS1 + inhibitor NC)

**Fig. 6:**
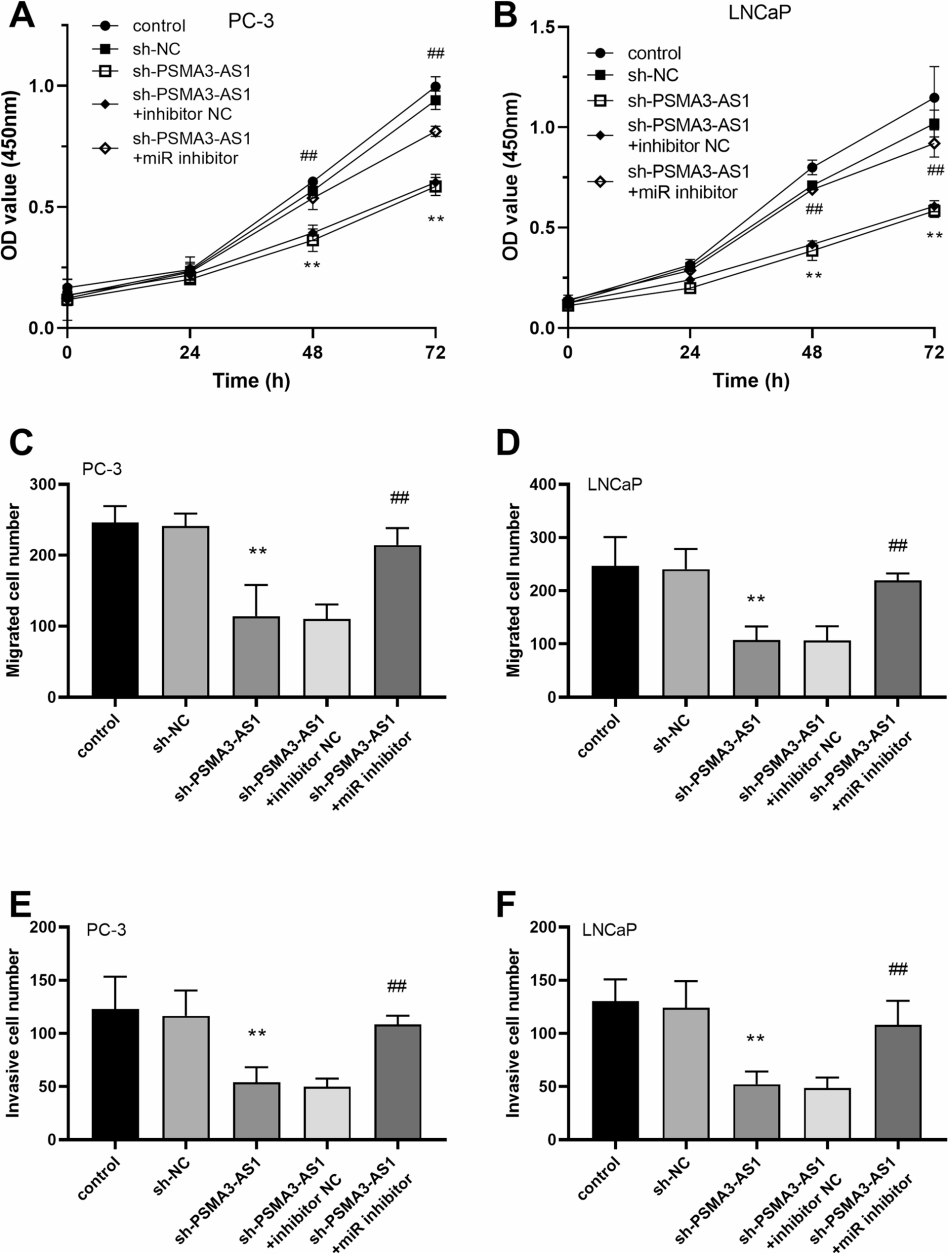
Effect of miR-29a-3p levels on PRAD cell lines. Inhibition of miR-29a-3p expression also reversed the decrease in proliferation, migration and invasion of LNCaP and PC-3 cells due to reduced PSMA3-AS1 expression levels **(A-F)**, (** *P* < 0.01 vs. sh-NC; ## *P* < 0.01 vs. sh- PSMA3-AS1 + inhibitor NC)

Expression levels of miR-29a-3p were markedly lower in all PRAD cell lines compared with RWPE-1 (Fig. 5A, *P* < 0.01). In LNCaP and PC-3 cells, knocking down PSMA3-AS1 significantly increased the expression level of miR-29a-3p. However, this effect was notably suppressed by adding a miR-29a-3p inhibitor (Fig. 5B-C, *P* < 0.01). Additionally, inhibiting the expression of miR-29a-3p reversed the decrease in the proliferative capacity of LNCaP and PC-3 cells caused by reduced PSMA3-AS1 expression level (Fig. 6A-B, *P* < 0.01). Inhibition of miR-29a-3p levels promoted the development of PRAD cell lines. The migratory and invasive abilities of PRAD cells that were reduced by PSMA3-AS1 were notably restored after inhibiting miR-29a-3p levels (Fig. 6C-F, *P* < 0.01). We predicted the downstream target genes of miR-29a-3p based on five databases (miRDB, miRWalk, TargetScan, ENCORI, miRPathDB), and identified a total of 270 overlapping target genes (Figure S1). Subsequently, PPI analysis was performed on these target genes and the top ten target genes with the highest node degree were listed, and the results are shown in Figure S2. Among them, the target gene with the highest node degree was STAT3.

## Discussion

PRAD is a malignant tumor of the urinary tract, and is the fifth leading cause of cancer-related death in men [24]. Epidemiological studies have shown that PRAD tends to develop after the age of 55 and increases linearly with age. Most patients are in an advanced stage of PRAD at the time of initial consultation, having missed the optimal treatment period, which leads to a poor prognosis [25]. However, effective prognostic biomarkers are lacking at this stage.

LncRNAs have the potential to become prognostic markers due to their specific expression in diseases [26]. LncRNA PSMA3-AS1 is specifically level in many diseases and has the potential to become a diagnostic or prognostic marker [27, 28]. The long-chain non-coding RNA PSMA3-AS1 functions as a competitive endogenous RNA and promotes gastric cancer progression by regulating the miR-329-3p/ALDOA axis [29]. In addition, the higher stability of lncRNAs in fluids provides new ideas and tools for non-invasive diagnosis [30–32]. Our study explored for the first time the expression of PSMA3-AS1 in PRAD patients. In the database, PSMA3-AS1 was highly expressed in PRAD, and our results indicate that its expression levels were also markedly elevated in cancer tissues. Further data revealed a correlation between PSMA3-AS1 levels and the five-year survival of PRAD patients after surgery. PRAD expressing high levels of PSMA3-AS1 had shorter 5-year survival. COX regression analysis also indicated that PSMA3-AS1 was an independent prognostic factor for PRAD. Additionally, Gleason grading, TNM and PSA were identified as independent prognostic factors for PRAD patients.

Although lncRNAs do not encode proteins, they can participate in important pathways in the cell growth cycle and regulate physiological activities such as cell proliferation and migration [33]. For example, LncRNA FOXD2-AS1 promotes OSCC cell growth, invasion and migration by regulating the MiR-185-5p/PLOD1/Akt/mTOR pathway [34]. The long-chain non-coding RNA PSMA3 antisense RNA 1 promotes cell proliferation, migration and invasion in pancreatic ductal adenocarcinoma by targeting microRNA-154-5p and positively regulating karyopherin subunit α 4 [35]. PSMA3-AS1 has been shown to regulate the proliferation, migration and invasion activities of cholangiocarcinoma cells [36], as well as promoting the growth of lung cancer cells [37]. Our findings show a similar trend: high PSMA3-AS1 expression promotes the viability of PRAD cell lines. Knocking down PSMA3-AS1 significantly reduces physiological activities such as cell proliferation and migration. This suggests that high levels of PSMA3-AS1 may be associated with the development and poor prognosis of PRAD, which suggests that PSMA3-AS1 could be a potential target for preventing the poor prognosis of PRAD.

MicroRNAs (miRNAs) are the downstream target genes of lncRNAs, which act as miRNA sponges and can regulate their expression levels [38, 39], thereby affecting disease development and the cell growth cycle [40, 41]. Disruption of miR-29a-3p has been observed in various tumor diseases, and it is expected to become an important new target for early diagnosis, efficacy detection, and recurrence monitoring [42]. In our study, we found that miR-29a-3p was poorly expressed in PRAD patients. Furthermore, numerous lncRNAs modulate cellular activities by regulating miR-29a-3p; for instance, LINC00852 modulates prostate cancer cell proliferation and invasion by regulating the MiR-29a-3p/JARID2 axis [43]. TUG1 promotes pre-eclampsia by regulating the miR-29a-3p/VEGFA pathway and the Ang2/Tie2 pathway in angiogenesis in HUVECs [44]. lncRNA HOXA-AS3 targets miR-29a-3p in cervical cancer for its prognostic value and its role in regulating tumor progression [45]. Our study validated for the first time the target-binding relationship between PSMA3-AS1 and miR-29a-3p and explored the effect of PSMA3-AS1 on PRAD cells via miR-29a-3p. Our results showed that the knockdown of lncRNA PSMA3-AS1 resulted in elevated levels of miR-29a-3p, which in turn affected reduced PRAD cell growth and migration. Therefore, regulating the PSMA3-AS1/miR-29a-3p axis may affect the development and prognostic status of PRAD. In addition, lncRNAs can be involved in regulating disease progression by modulating miRNA expression levels and thus altering mRNA/protein expression or content. For example, LncRNA KCNQ1OT1 promotes NLRP3 inflammasome activation in Parkinson’s disease by regulating the pri-miR-186/mature miR-186/NLRP3 axis [46]. We predicted the downstream target genes of miR-29a-3p based on five databases (miRDB, miRWalk, TargetScan, ENCORI, miRPathDB), and a total of 270 overlapping target genes were identified (Figure S1). Subsequently, these target genes were analyzed by PPI and listed as the top ten target genes in terms of node degree, and the results are shown in Figure S2. Among them, the target gene with the highest node degree was STAT3. Some studies have reported that STAT3 is crucial in the occurrence and development of PRAD. Restoration of miR-26a-5p by EZH2 silencing can block the IL-6/STAT3 axis to inhibit prostate cancer growth [47]. Therefore, we hypothesized that the expression level of miR-29a-3p could be promoted by silencing PSMA3-AS1, thereby blocking the role of STAT3 in PRAD and delaying the development of PRAD. It has also been reported that the mitotic DNA integrity checkpoint signaling pathway is associated with cancer, and that it may be involved in regulating genome stability in cancer [48]. And the relationship between STAT3 and this pathway needs to be further investigated.

However, there are some limitations in our study: firstly, the role of PSMA3-AS1/miR-29a-3p/STAT3 axis in PRAD needs to be further confirmed by further analyses of signaling pathways or transcriptomics. The existing experimental results need to be validated by more experimental models (e.g., animal models); second, the safety of PSMA3-AS1 in clinical applications needs to be explored. Finally, we will study non-invasive detection of PSMA3-AS1 in more depth, with the aim of providing a new theoretical basis for the non-invasive diagnosis of PRAD. In addition, the Kaplan Meier survival curve analyses of survival in the PSMA3-AS1 high and low expression groups have some limitations, including their reliance on the assumption of proportional risk and the possible bias of meta-analyses using pooled statistics. These include their reliance on the assumption of proportional risk and the possibility of bias in meta-analyses using summary statistics. Therefore, we will improve this method of analysis in subsequent experiments, referring to the articles by Liu et al. [49–51], to improve the survival analysis method.

Overall, the level of PSMA3-AS1 expression was high in the cancer tissues of PRAD patients, while the level of expression of miR-29a-3p was low. A high level of PSMA3-AS1 was significantly correlated with a poor prognosis for PRAD patients, suggesting its potential as a prognostic biomarker. In addition, the level of miR-29a-3p could be increased by knocking down PSMA3-AS1, which slowed the proliferation, migration and invasion of cancer cells. Therefore, regulating the expression level of the PSMA3-AS1/miR-29a-3p axis could influence the development of PRAD.

## Electronic supplementary material

Below is the link to the electronic supplementary material.

Supplementary Material 1

Supplementary Material 2

## Data Availability

The datasets used and/or analysed during the current study are available from the corresponding author on reasonable request.

## References

[1] Sonkin D, Thomas A, Teicher BA. Cancer treatments: past, present, and future. Cancer Genet. 2024; 286–28718. 10.1016/j.cancergen.2024.06.002 PMC11338712 38909530

[2] Wang C, Dong Q, Liu X, Ni M, Xie Q, Xiao J, et al. Protocol for SNOTOB study: radical prostatectomy without prostate biopsy following (18)F-PSMA-1007 PET/CT based on a diagnostic model: a single-centre, single-arm, open-label study. BMJ Open. 2023;13(11):e073983. 37984956 10.1136/bmjopen-2023-073983 PMC10660686

[3] Yang F, Qin Q, Liu J. Predictive value of microRNA-133a-3p for early urinary incontinence after radical prostatectomy for prostate cancer and its correlation with rehabilitation effects. Hereditas. 2025;162(1):75. 40350457 10.1186/s41065-025-00443-2 PMC12067700

[4] Yin L, Yue C, Jing H, Yu H, Zuo L, Liu T. No association between three polymorphisms (rs1800629, rs361525 and rs1799724) in the tumor necrosis factor-α gene and susceptibility to prostate cancer: a comprehensive meta-analysis. Hereditas. 2020;157(1):11. 32264962 10.1186/s41065-020-00125-1 PMC7137332

[5] Chauhan K, Ebner DK, Tzou K, Ryan K, May J, Kaleem T et al. Assessment of bladder filling during prostate cancer radiation therapy with ultrasound and cone-beam CT. Front Oncol. 2023; 131200270. 10.3389/fonc.2023.1200270 PMC10426376 37588094

[6] Liu Z, Kuang S, Chen Q. A review focusing on the role of pyroptosis in prostate cancer. Med (Baltim). 2023;102(50):e36605. 10.1097/MD.0000000000036605 PMC10727670 38115248

[7] Jomy J, Sharma R, Lu R, Chen D, Ataalla P, Kaushal S et al. Clinical impact of radiotherapy quality assurance results in contemporary cancer trials: a systematic review and meta-analysis. Radiother Oncol. 2025; 207110875. 10.1016/j.radonc.2025.110875 40185159

[8] Sun Y, Gong J, Li Z, Han L, Sun D. Gallbladder cancer: surgical treatment, immunotherapy, and targeted therapy. Postgrad Med. 2024;136(3):278–91. 38635593 10.1080/00325481.2024.2345585

[9] Gao S, Xu T, Wang W, Li J, Shan Y, Wang Y, et al. Polysaccharides from anemarrhena asphodeloides bge, the extraction, purification, structure characterization, biological activities and application of a traditional herbal medicine. Int J Biol Macromol. 2025;311(Pt 1):143497. 40286959 10.1016/j.ijbiomac.2025.143497

[10] Khan SR, Breadner D. Unveiling the synergistic potential: bispecific antibodies in conjunction with chemotherapy for advanced Non-Small-Cell lung Cancer treatment. Curr Oncol. 2025; 32(4). 10.3390/curroncol32040206 PMC12025875 40277763

[11] Ma Z, Liu X, Zhang X, Li S, An J, Luo Z. Research progress on long non–coding RNAs in non–infectious spinal diseases (Review). Mol Med Rep. 2024; 30(3). 10.3892/mmr.2024.13288 PMC11267249 38994759

[12] Dong X, Cong S. The emerging roles of long non-coding RNAs in polyglutamine diseases. J Cell Mol Med. 2021;25(17):8095–102. 34318578 10.1111/jcmm.16808 PMC8419158

[13] Zhou C, Qiu Q, Liu X, Zhang T, Liang L, Yuan Y, et al. Novel exosome-associated LncRNA model predicts colorectal cancer prognosis and drug response. Hereditas. 2025;162(1):79. 40380258 10.1186/s41065-025-00445-0 PMC12082951

[14] Li Y, Wei C, Huang C, Ling Q, Zhang L, Huang S, et al. Long noncoding RNA as a potential diagnostic tool for prostate cancer: a systematic review and meta-analysis. Biomarkers. 2023;28(1):1–10. 36323640 10.1080/1354750X.2022.2142293

[15] Cao X, Luan K, Yang J, Huang Y. Targeting LncRNA PSMA3-AS1, a prognostic marker, suppresses malignant progression of oral squamous cell carcinoma. Dis Markers. 2021; 20213138046. 10.1155/2021/3138046 PMC8397548 34457087

[16] Syllaios A, Gazouli M, Vailas M, Mylonas KS, Sakellariou S, Sougioultzis S et al. The expression patterns and implications of MALAT1, MANCR, PSMA3-AS1 and miR-101 in esophageal adenocarcinoma. Int J Mol Sci. 2023; 25(1). 10.3390/ijms25010098 PMC10778904 38203269

[17] Cheng G, Li Y, Liu Z, Song X. LncRNA PSMA3-AS1 promotes the progression of non-small cell lung cancer through targeting miR-17-5p/PD-L1. Adv Clin Exp Med. 2021;30(10):1043–50. 34610219 10.17219/acem/138624

[18] Peng P, Wang Y, Wang BL, Song YH, Fang Y, Ji H, et al. LncRNA PSMA3-AS1 promotes colorectal cancer cell migration and invasion via regulating miR-4429. Eur Rev Med Pharmacol Sci. 2020;24(22):11594–601. 33275226 10.26355/eurrev_202011_23802

[19] Retraction. Long non-coding RNA PSMA3-AS1 enhances cell proliferation, migration and invasion by regulating miR-302a-3p/RAB22A in glioma. Biosci Rep. 2023; 43(3). 10.1042/BSR-2019-1571_RET PMC9977714 36856005

[20] Zhang M, Xu Y, Yin S, Qiu F. YY1-induced long non-coding RNA PSMA3 antisense RNA 1 functions as a competing endogenous RNA for MicroRNA 214-5p to expedite the viability and restrict the apoptosis of bladder cancer cells via regulating programmed cell death-ligand 1. Bioengineered. 2021;12(2):9150–61. 34720049 10.1080/21655979.2021.1994907 PMC8809964

[21] Yin G, Guo W, Wang R, Li N, Chen X, Zhang Y, et al. Analysis of the role of IL-1 family and related genes in head and neck squamous cell carcinoma. Braz J Otorhinolaryngol. 2025;91(1):101484. 39461030 10.1016/j.bjorl.2024.101484 PMC11543642

[22] Xiao S, Lou W. Integrated analysis reveals a potential cuproptosis-related CeRNA axis SNHG17/miR-29a-3p/GCSH in prostate adenocarcinoma. Heliyon. 2023;9(11):e21506. 38027603 10.1016/j.heliyon.2023.e21506 PMC10651496

[23] Kong Z, Wan X, Lu Y, Zhang Y, Huang Y, Xu Y, et al. Circular RNA circFOXO3 promotes prostate cancer progression through sponging miR-29a-3p. J Cell Mol Med. 2020;24(1):799–813. 31733095 10.1111/jcmm.14791 PMC6933405

[24] Zhu W, Liu H, Wang X, Lu J, Zhang H, Wang S et al. Associations of CYP1 polymorphisms with risk of prostate cancer: an updated meta-analysis. Biosci Rep. 2019; 39(3). 10.1042/BSR20181876 PMC6395298 30765615

[25] Han J, Zhou Y, Zhang C, Feng J, Wang J, Guo K, et al. Intratumoral immune heterogeneity of prostate cancer characterized by typing and hub genes. J Cell Mol Med. 2023;27(1):101–12. 36524848 10.1111/jcmm.17641 PMC9806298

[26] Han SH, Ko JY, Kang ES, Park JH, Yoo KH. Long non-coding rnas: key regulators of liver and kidney fibrogenesis. BMB Rep. 2023;56(7):374–84. 37357534 10.5483/BMBRep.2023-0075 PMC10390290

[27] Yang J, Liu K, Chen L, He H, Li T, Li L et al. Revisiting the role of long Non-coding RNA PSMA3-AS1 in human cancers: current evidence and future directions. Curr Pharm Des. 2025. 10.2174/0113816128350406241223053744 39901687

[28] Peng M, Yuan H. LncRNA PSMA3-AS1 activates the progression of triple-negative breast cancer cells by blocking miR-186-5p-mediated PSME3 Inhibition. Cell Mol Biol (Noisy-le-grand). 2023;69(14):81–7. 38279473 10.14715/cmb/2023.69.14.13

[29] Kan L, Yang M, Zhang H. Long noncoding RNA PSMA3-AS1 functions as a competing endogenous RNA to promote gastric cancer progression by regulating the miR-329-3p/ALDOA axis. Biol Direct. 2023;18(1):36. 37403106 10.1186/s13062-023-00392-8 PMC10318671

[30] Jahangiri L. Updates on liquid biopsies in neuroblastoma for treatment response, relapse and recurrence assessment. Cancer Genet. 2024; 288–28932. 10.1016/j.cancergen.2024.09.001 39241395

[31] Ohyama H, Hirotsu Y, Amemiya K, Mikata R, Amano H, Hirose S et al. Development of a molecular barcode detection system for pancreaticobiliary malignancies and comparison with next-generation sequencing. Cancer Genet. 2024; 280–2816. 10.1016/j.cancergen.2023.12.002 38113555

[32] Gonzalez T, Nie Q, Chaudhary LN, Basel D, Reddi HV. Methylation signatures as biomarkers for non-invasive early detection of breast cancer: A systematic review of the literature. Cancer Genet. 2024; 282–2831. 10.1016/j.cancergen.2023.12.003 38134587

[33] Wang X, Zhang R, Wu S, Shen L, Ke M, Ouyang Y, et al. Super-Enhancer LncRNA LINC00162 promotes progression of bladder Cancer. iScience. 2020;23(12):101857. 33344916 10.1016/j.isci.2020.101857 PMC7736918

[34] Liu J, Zhang Y, Wu J, Liu X, Li L, Zhang J. LncRNA FOXD2-AS1 promotes the growth, invasion and migration of OSCC cells by regulating the MiR-185-5p/PLOD1/Akt/mTOR pathway. Cancer Genet. 2024; 284–28548. 10.1016/j.cancergen.2024.05.001 38729078

[35] Bi J, Liang W, Wang Y, Tian W, Cao S, Liu P, Long Noncoding. RNA PSMA3 antisense RNA 1 promotes cell proliferation, migration, and invasion in pancreatic ductal adenocarcinoma via targeting MicroRNA-154-5p to positively modulate Karyopherin subunit alpha 4. Pancreas. 2022;51(8):1037–46. 36607951 10.1097/MPA.0000000000002136

[36] Sun D, Li F, Liu L, Yu S, Wang H, Gao X, et al. PSMA3-AS1 induced by transcription factor PAX5 promotes cholangiocarcinoma proliferation, migration and invasion by sponging miR-376a-3p to up-regulate LAMC1. Aging. 2022;14(1):509–25. 35022330 10.18632/aging.203828 PMC8791211

[37] Li F, Yu L, Zhu J. LncRNA PSMA3-AS1 promotes lung Cancer growth and invasion via sponging MiR-4504. Cancer Manag Res. 2020; 125277–83. 10.2147/CMAR.S253575 PMC7335846 32669876

[38] Li X, Lv X, Li Z, Li C, Li X, Xiao J et al. Long noncoding RNA ASLNC07322 functions in VEGF-C expression regulated by Smad4 during Colon cancer metastasis. Mol Ther Nucleic Acids. 2019; 18851–62. 10.1016/j.omtn.2019.10.012 PMC6861657 31739210

[39] Yu L, Chen D, Song J. LncRNA SNHG16 promotes non-small cell lung cancer development through regulating EphA2 expression by sponging miR-520a-3p. Thorac Cancer. 2020;11(3):603–11. 31953899 10.1111/1759-7714.13304 PMC7049505

[40] Guo Y, Liu Y, Wang H, Liu P. Long noncoding RNA SRY-box transcription factor 2 overlapping transcript participates in parkinson’s disease by regulating the microRNA-942-5p/nuclear apoptosis-inducing factor 1 axis. Bioengineered. 2021;12(1):8570–82. 34607512 10.1080/21655979.2021.1987126 PMC8806952

[41] Chen Y, Zheng J, Su L, Chen F, Zhu R, Chen X et al. Increased Salivary microRNAs That Regulate DJ-1 Gene Expression as Potential Markers for Parkinson’s Disease. Front Aging Neurosci. 2020; 12210. 10.3389/fnagi.2020.00210 PMC7360355 32733234

[42] He D, Wang H, Ho SL, Chan HN, Hai L, He X, et al. Total internal reflection-based single-vesicle in situ quantitative and stoichiometric analysis of tumor-derived Exosomal MicroRNAs for diagnosis and treatment monitoring. Theranostics. 2019;9(15):4494–507. 31285775 10.7150/thno.33683 PMC6599656

[43] Zhang H, Du Y, Xin P, Man X. The LINC00852/miR-29a-3p/JARID2 axis regulates the proliferation and invasion of prostate cancer cell. BMC Cancer. 2022;22(1):1269. 36471281 10.1186/s12885-022-10263-6 PMC9724404

[44] Liu Z, Guo N, Zhang XJ. Long noncoding TUG1 promotes angiogenesis of HUVECs in PE via regulating the miR-29a-3p/VEGFA and Ang2/Tie2 pathways. Microvasc Res. 2022; 139104231. 10.1016/j.mvr.2021.104231 34352236

[45] Xu H, Tang Y, He C, Tian Y, Ni R. Prognostic value of LncRNA HOXA-AS3 in cervical cancer by targeting miR-29a-3p and its regulatory effect on tumor progression. J Obstet Gynaecol Res. 2022;48(10):2594–602. 35817473 10.1111/jog.15360 PMC13469931

[46] Li MM, Shi MJ, Feng CC, Yu ZY, Bai XF, Lu L. LncRNA KCNQ1OT1 promotes NLRP3 inflammasome activation in parkinson’s disease by regulating pri-miR-186/mature miR-186/NLRP3 axis. Biochim Biophys Acta Mol Basis Dis. 2024;1870(8):167454. 39122224 10.1016/j.bbadis.2024.167454

[47] Zhang W, Nie Q, Zhang X, Huang L, Pang G, Chu J, et al. miR-26a-5p restoration via EZH2 Silencing blocks the IL-6/STAT3 axis to repress the growth of prostate cancer. Expert Opin Ther Targets. 2023;27(12):1285–97. 38155599 10.1080/14728222.2023.2293750

[48] Rasteh AM, Liu H, Wang. Pan-cancer genetic profiles of mitotic DNA integrity checkpoint protein kinases. Cancer Biomark. 2024;41(3–4):Cbm240119. 40095483 10.3233/CBM-240119 PMC11905933

[49] Liu H, Weng J, Huang CL, Jackson AP. Is the voltage-gated sodium channel β3 subunit (SCN3B) a biomarker for glioma? Funct Integr Genomics. 2024;24(5):162. 39289188 10.1007/s10142-024-01443-7

[50] Liu H, Tang T. MAPK signaling pathway-based glioma subtypes, machine-learning risk model, and key hub proteins identification. Sci Rep. 2023;13(1):19055. 37925483 10.1038/s41598-023-45774-0 PMC10625624

[51] Liu H, Dong A, Rasteh AM, Wang P, Weng J. Identification of the novel exhausted T cell CD8 + markers in breast cancer. Sci Rep. 2024;14(1):19142. 39160211 10.1038/s41598-024-70184-1 PMC11333736

