# Supplementary figures and images for: Prognostic value of LncRNA PSMA3-AS1 in prostate cancer and its potential regulatory mechanism
Source: Hereditas. 2025 Jul 12;162:127. doi: 10.1186/s41065-025-00485-6 (PMC12255973; doi:10.1186/s41065-025-00485-6)

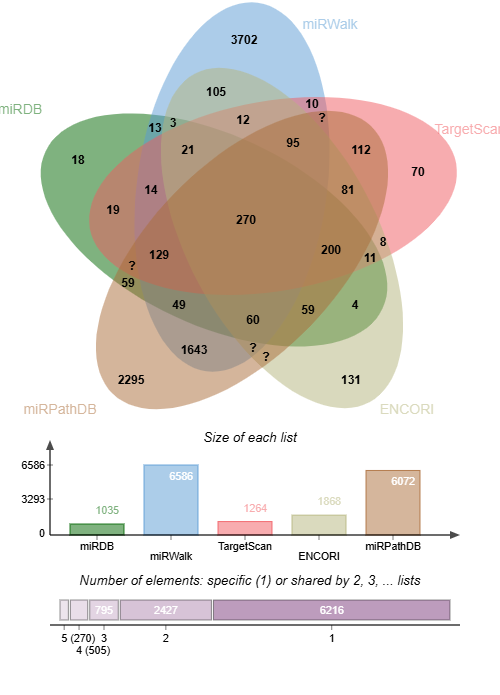

Supplement: Supplementary file 1 — Supplementary Material 1 [file 41065_2025_485_MOESM1_ESM.png]
